# A rare huge retroperitoneal accessory spleen mimicking right-sided sarcoma in a female patient: case report and review

**DOI:** 10.1093/jscr/rjaf024

**Published:** 2025-02-05

**Authors:** Hassan Al-Thani, Ayman El-Menyar, Yaser M Ata, Lana Abu Afifeh, Sali Alatasi, Hussam Telfah, Maryam Al-Sulaiti, AbdelHakem Tabeb

**Affiliations:** Department of Surgery, Trauma and Vascular Surgery, Hamad Medical Corporation, Al Rayyan Road, PO Box 3050, Doha, Qatar; Department of Surgery, Trauma and Vascular Surgery, Hamad Medical Corporation, Al Rayyan Road, PO Box 3050, Doha, Qatar; Department of Clinical Medicine, Weill Cornell Medicine, PO Box 24144, Doha, Qatar; Department of Surgery, Urology, Hamad Medical Corporation, Doha, Qatar; Department of Surgery, General Surgery, Hamad Medical Corporation, Doha, Qatar; Department of Histopathology, Hamad Medical Corporation, Doha, Qatar; Department of Histopathology, Hamad Medical Corporation, Doha, Qatar; Department of Surgery, General Surgery, Hamad Medical Corporation, Doha, Qatar; Department of Surgery, General Surgery, Hamad Medical Corporation, Doha, Qatar

**Keywords:** retroperitoneal, accessory spleen, intraperitoneal, abdominal, organ, spleniculi

## Abstract

Detecting retroperitoneal accessory spleen (AS) requires a high index of suspicion for proper and timely diagnosis. The AS can be found near the hilum of the spleen or wholly or partially embedded in the pancreatic tail, stomach, bowel, mesentery walls, or even in the pelvis. Left-sided retroperitoneal AS is common compared to the right-sided retroperitoneal location, which is very rare. The diagnosis of AS is not common preoperatively when investigating a mass in the other abdominal regions, and the surgical resection can thoroughly confirm the diagnosis. The management of AS is surgical excision through open, laparoscopic, or robotic-assisted techniques, depending on the patient status, the size and location of the mass, and the available treatment modality in the hospital. Here, we described a rare case presentation of a huge right retroperitoneal mass (AS) that was initially suspected as sarcoma and managed by laparotomy resection.

## Introduction

The spleen is an intraperitoneal mesodermal in origin derived from dorsal mesentery as a condensation of mesodermal mesenchyme [1]. The splenic artery is often a branch of the celiac trunk, but, in very rare conditions, it arises from the superior mesenteric artery (<1%) [2]. The splenic vein, the main draining vessel for the spleen, unites with the inferior mesenteric vein and then joins the superior mesenteric vein to form the portal vein [3].

A few congenital splenic anomalies have been described including accessory spleen (AS) and splenogonadal fusion. The AS is a congenital defect with an additional splenic tissue to the native spleen due to an incomplete fusion of splenic masses during the embryologic period [4]. The retroperitoneal location of the AS is rare condition that needs a high index of suspicion to avoid complications (torsion, rupture, hematological abnormalities, hypertrophy, and compression) and unnecessary surgical intervention in asymptomatic small lesions. AS should be differentiated from an ectopic spleen [4].

Here we present a rare case of a huge right-sided retroperitoneal AS that was preoperatively diagnosed as retroperitoneal sarcoma.

## Case presentation

A 40-year-old female nulliparous had an incidental radiological finding of a large retroperitoneal mass alongside multiple uterine fibroids. She was diagnosed with a right abdominal pain for 1 year, radiating to the lower back. The pain was constantly mild at the right side of the abdomen aggravated by walking and relieved with analgesics. Recently, the pain became progressive. No associated urinary or other gastrointestinal symptoms were reported, with no remarkable medical or surgical history.

Physical examination revealed large palpable abdominal mass on the right side extending to the umbilicus. Laboratory blood results were unremarkable. Ultrasonography examination demonstrated an 8.9 × 8.2 cm right adnexal cystic structure of echogenic content and internal vascularity. The right ovary was not seen separately; however, a stretched ovarian tissue is likely appreciated in the periphery of right adnexal lesion and mesenteric lymph node was noted in the right iliac fossa.

**Figure 1:**
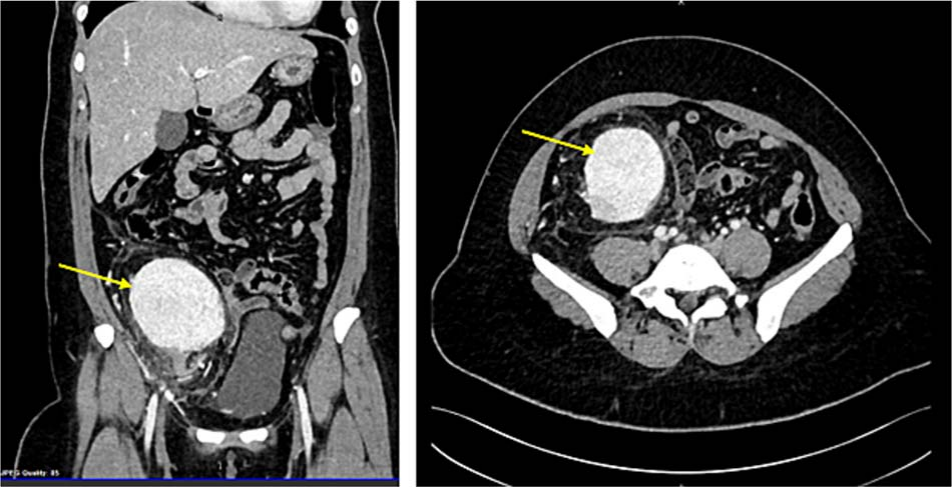
CT abdomen demonstrating the enlarged mass and its position by sagital and coronal sections (Arrow points at the mass).

**Figure 2:**
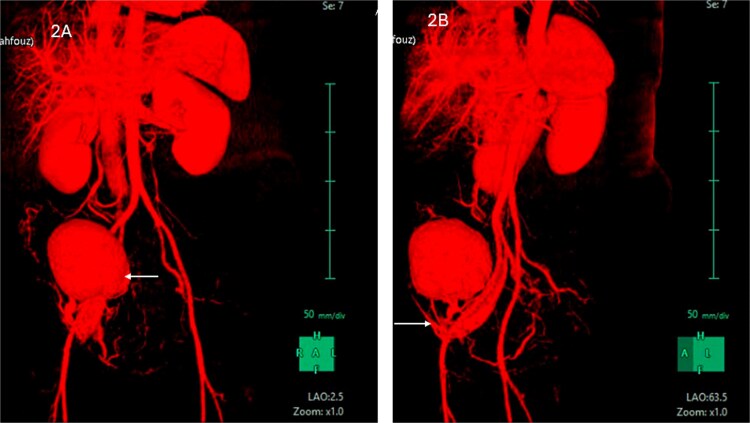
(A) CT abdomen with contrast demonstrating the mass in the right iliac fossa. (B) The image is rotated to show the blood supply from right external iliac artery and vein.

CT scan revealed evidence of large right iliac fossa soft tissue mass, measuring 8.5 × 8.2 × 8.4 cm, displacing the adjacent abdominal and pelvic organs with significant peri-lesional fat strandings. The mass shows homogenous hyper enhancement in post contrast with peripheral slight enhancing area [3.3 × 1.8 cm] with adjacent bowel loops tethering with peritoneal thickening. There were multiple adjacent para-aortic, right external iliac, and common iliac lymph nodes enlargement (Fig. 1). The mass vessels were arising from the right external iliac artery and vein (Fig. 2).

**Figure 3:**
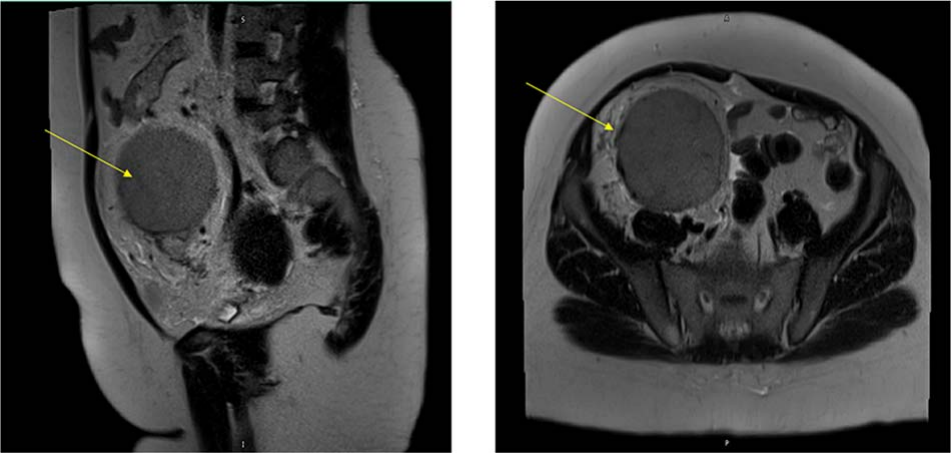
MRI abdomen signifying the mass and its location, extent in both views (Arrow points at the mass).

The MRI showed a large right iliac fossa extraperitoneal soft tissue mass, measuring 8.3 × 2.2 × 8.4 cm. The mass shows intermediate T2, low T1, and marked diffusion restriction. Dynamic imaging shows avid early contrast enhancement with retention of contrast in delayed imaging. The original spleen was in its normal place. The uterus showed multiple small intramural and subserosal fibroids with a low T2 signal, and an isointense T1 signal with no diffusion restriction or suspicious enhancement (Fig. 3).

**Figure 4:**
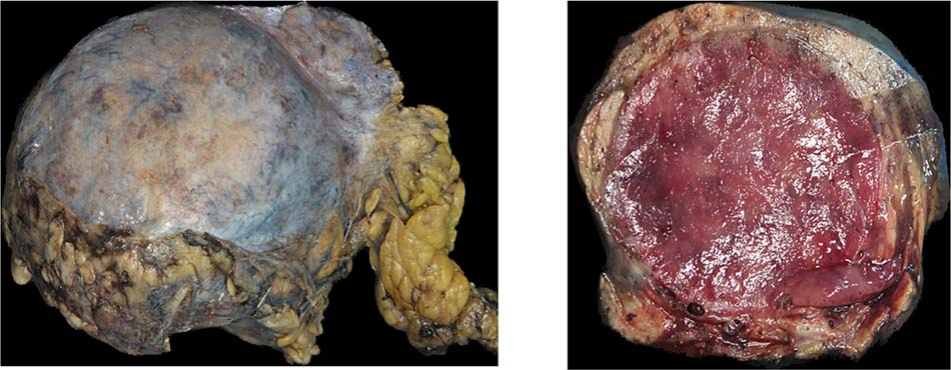
(A) Gross examination picture of retroperitoneal mass measuring. (B) Gross examination picture of the mass cut surfaces shows a cherry red and nodular texture.

**Figure 5:**
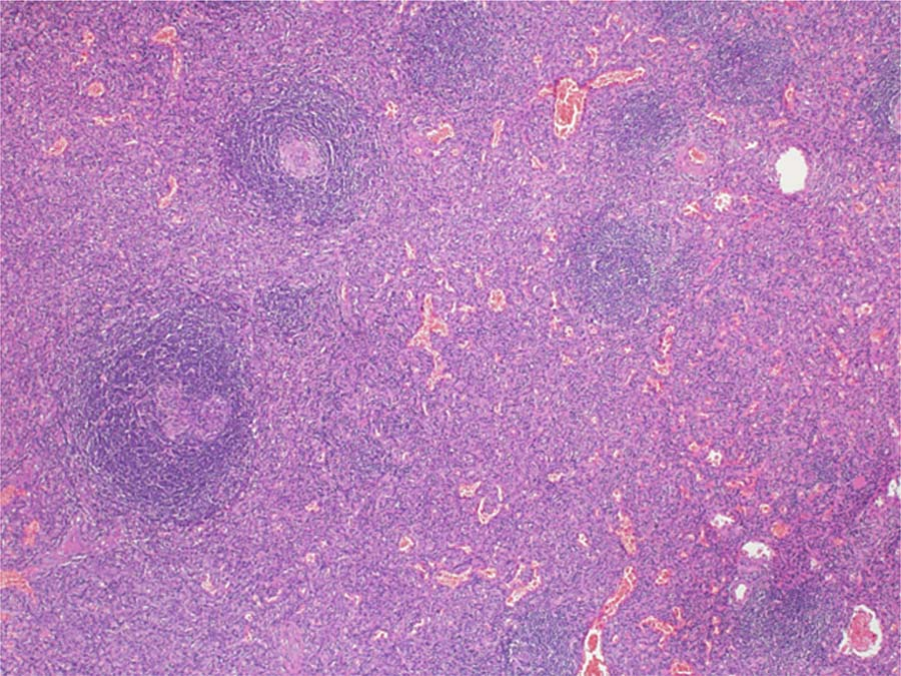
Microscopic examination picture confirmed the splenic tissue that included white and red pulps (hematoxylin & eosin stain 40×).

The patient underwent laparotomy for a planned excision of retroperitoneal mass as if it was a sarcoma. A temporary bilateral ureteral stent was placed. The mass was extending up from the right side of the retroperitoneal abdomen all the way to the right iliac fossa and pelvis. The mass was adherent to the common iliac artery and external iliac artery. Dissection was carried out using sharp and blunt dissection as well as the Harmonic scalpel. The blood supply from the external iliac artery was ligated and venous drain was sutured ligated. Because multiple large pedunculated fibroids (measuring 8 × 7 cm the largest, and the second one was 5 × 5 cm, there was one 3 × 3 cm, there were another two smaller ones, measuring 3 × 2 cm and one small 1 cm), excision of the three largest fibroids facilitate for a complete excision of the retroperitoneal mass (Fig. 4) and was sent for histopathology, which exhibited retroperitoneal AS (Fig. 5) negative for malignancy. The patient had an uneventful hospital course and was discharged 1 week later.

## Discussion

There are two types of AS, intraperitoneal and retroperitoneal. The intraperitoneal is further subdivided based on the size of the splenic tissue (AS and spleniculi) [5]. On the other hand, retroperitoneal spleen subdivided based on the location (right- and left-sided retroperitoneal spleen). The intraperitoneal AS is generally an asymptomatic small mass of <1 cm, but it can reach up to 3–10 cm in a few cases and receives its blood supply from the splenic artery branches [4, 6]. AS is usually well capsulated and does not have fixed location as it was found near the hilum of the spleen (the most common site) or wholly or partially embedded in the tail of the pancreas, stomach wall, bowel, or mesentery wall and even in the pelvis [7]. Generally, the retroperitoneal AS is uncommon, but the left-sided retroperitoneal AS is common compared to the right-sided retroperitoneal location, which is very rare [2–4, 6, 8, 9]. Zouaghi *et al*. reported a left-sided retroperitoneal AS of 10 cm in a female patient diagnosed with left lower quadrant pain [4]. The mass was receiving its blood supply directly from the aorta and was diagnosed as a vascular tumor preoperatively. Grochowska *et al*. reported nine cases with rupture of an AS following blunt trauma [10]. The authors suggested that the presence of AS could be beneficial following traumatic splenic rupture because the AS can replace the function of the primary spleen.

Intraperitoneal AS is a relatively common condition and easily mistaken for a neoplasm preoperatively. It is found incidentally during abdominal imaging, and it comprises an incidence rate between 10 and 30% at autopsy. It is more common in females and often found as a single nodule, or multiple lesions with various sizes with oval or triangular shapes and histologically composed of red and white pulps as the primary spleen [5, 7, 10].

AS is not decisively diagnosed radiologically including ultrasound, CT-scan, or MRI as these modalities cannot differentiate the tissue accurately, although previous studies suggested that Radionuclide imaging using Tc99m labeled red blood cells is considered the diagnostic modality of choice because the RBC labeled with TC 99m is taken from the reticulum-endothelium and appears in the spleen, liver, and bone marrow. However, it is not done frequently as the suspicious of AS is not common when investigating a mass in the other abdominal regions and the surgical resection of the mass can thoroughly confirm the diagnosis [6, 11–14].

The management option for AS is surgical excision through open, laparoscopic, or robotic-assisted techniques depending on the patient status, the size and location of the mass, and the available modality in the hospital. In our case, due to the suspicion of sarcoma, the size and location of the mass laparotomy were done, and complete oncological resection of the mass and the adjacent lymph nodes was secured and sent for histopathologic investigation. Table 1 summarizes five reported cases of AS including ours [5, 8, 9, 15].

**Table 1:** Review of literature of cases of right-side retroperitoneal AS

Author, year	Age (years), gender	Presentation	Imaging	Size (cm)	Blood supply	Location	Preoperative working diagnosis	Management
Kim *et al*., 2008 [9]	68, male	Incidental	US, CT, MRI	4 × 3.8	Not reported	Right suprarenal space	Retroperitoneal tumor	Laparoscopic
Arra *et al*., 2013 [15]	24, male	Abdominal mass		20	Originate from the aorta and drain into the inferior vena cava	Right suprarenal region	Malignant adrenal tumor	Open surgery
Zhou *et al*., 2015 [8]	40, female	Incidental	US, CT, MRI	3.4 × 2.5	Not reported	Right retroperitoneal region	Neoplasm	Retroperitoneoscopic
Maharaj *et al*., [5]	44, female	Right upper quadrant pain	US, CT	11 × 8	Arterial supply not reported drain to inferior vena cava	Retro-duodenal and lying just anterior to the right kidney	Duodenal gastro-intestinal stromal tumor and a retroperitoneal sarcoma	Open surgery
Current case	40, female	Incidental	US, CT, MRI	8.9 × 8.2	Supply from right external iliac artery and vein	Right iliac fossa, retroperitoneal	Sarcoma	Open surgery

## Conclusion

This case report describes a rare presentation of a huge right retro-peritoneal mass that was managed by laparotomy resection. The diagnosis was histo-pathologically as an AS. AS should be suspected as a differential diagnosis of any retroperitoneal masses regardless of its size.

## Data Availability

All data generated or analyzed during this study are included in this article. Further inquiries can be directed to the corresponding author.
